# Neoadjuvant Immunotherapy of Oral Squamous Cell Carcinoma: Case Report and Assessment of Histological Response

**DOI:** 10.3389/fonc.2021.720951

**Published:** 2021-07-21

**Authors:** Manuel Olmos, Jacek Glajzer, Tjark-Ole Büntemeyer, Gesche Frohwitter, Jutta Ries, Markus Eckstein, Markus Hecht, Rainer Lutz, Marco Rainer Kesting, Manuel Weber

**Affiliations:** ^1^ Department of Oral and Maxillofacial Surgery, Friedrich-Alexander University Erlangen-Nürnberg (FAU), Erlangen, Germany; ^2^ Comprehensive Cancer Center Erlangen-EMN (CCC ER-EMN), Erlangen, Germany; ^3^ Institute of Pathology, Friedrich-Alexander University Erlangen-Nürnberg (FAU), Erlangen, Germany; ^4^ Department of Radiation Oncology, Friedrich-Alexander University Erlangen-Nürnberg (FAU), Erlangen, Germany

**Keywords:** case report, immunotherapy, oral cancer, pembrolizumab, neoadjuvant, PD-1 - PD-L1 axis, OSCC, immune checkpoint inhibitors

## Abstract

**Background:**

The treatment of oral cancer remains challenging due to its infiltrative nature and a high tendency for tumour relapse leading to an overall poor prognosis. In the case of early recurrence, the patient’s prognosis deteriorates dramatically, with survival rate dropping to below 30%. Minimal improvements in survival trends in recurrent and advanced stage tumours have been reported in recent decades. Neoadjuvant immunotherapy may represent a new therapeutic approach changing the standard of care in advanced oral cancer therapy.

**Case Presentation:**

We describe the case of a woman in her late 30’s who presented in mid-2019 with oral squamous cell carcinoma (OSCC) localized to the floor of the mouth. After initial R0 resection, selective neck dissection, and adjuvant brachytherapy, an early recurrence of OSCC located between the hyoid bone and the mandible was diagnosed at the end of 2019. An off-label treatment regimen was performed with neoadjuvant use of Pembrolizumab 19 days prior to salvage surgery. Radiological and histological assessment of T-cell and programmed cell death protein 1 ligand 1 (PD-L1) expression was performed before and after checkpoint inhibitor application. Neoadjuvant immunotherapy resulted in increased T-cell infiltration and PD-L1 expression, as well as a significant tumour necrosis rate. One cycle of Pembrolizumab led to significant regressive tumour changes with increases in immune infiltration, sclerosis, and necrosis of 75% of the tumour mass with only 25% vital tumour cells remaining. By June 2020, the patient remained without recurrence.

**Conclusions:**

The case presented outlines the potential effects of neoadjuvant immunotherapy in recurrent or advanced OSCC prior to definitive surgical tumour treatment. The benefit of additional adjuvant treatment after histologic response will be discussed. The case is also analysed considering ongoing clinical trials of neoadjuvant immunotherapy for head and neck malignancies.

## Introduction

Oral cancer is a serious and growing problem in many parts of the globe including Europe. Oral and oropharyngeal cancer grouped together is the sixth most common cancer in the world (1). Considering the different entities within oral cancer, oral squamous cell carcinoma (OSCC) accounts for 92-95% of oral malignancies (2). Tumour recurrence is one of the main factors for the poor prognosis of OSCC, with the mortality rate increasing to 87% when early tumour recurrence is diagnosed (3). Current concepts on the aetiology of OSCC do not explain the occurrence of OSCC in young people without genetic conditions and a history without evidence of chemical noxious agents.

To date, the standard of care is primary surgical tumour resection with concurrent reconstruction and cervical lymph node dissection followed by postoperative adjuvant therapy (radiotherapy ± cisplatin) in high-risk cases (4). Current research focuses on the tumour-immune interaction in this regard. Common mechanisms of tumour immune evasion include loss of major histocompatibility complex (MHC) molecules and upregulation of immune checkpoint molecules on cell surfaces that normally regulate the amplitude and duration of a T-cell response (5, 6). The emerging treatment modality of immunotherapy targets immune checkpoint molecules including the programmed cell death protein 1 (PD-1) receptor and its ligand PD-L1 (7). Immune checkpoint blockade uses antibodies to block inhibitory immune checkpoint pathways and can invigorate inactive and/or exhausted T-cells to produce antitumor effects that confer long-term survival benefits in certain types of cancer (6, 8). Immunotherapy is a therapy for OSCC in cases with no curative surgical and radio-oncologic treatment option (9, 10). Neoadjuvant immunotherapeutic treatment of OSCC is currently not approved in the EU and US but is under intense investigation in large clinical trials (NCT02296684, NCT02641093 and NCT03765918). A recently published Phase 2 trial characterized neoadjuvant administration of Pembrolizumab 200 mg 2-3 weeks prior to surgery as safe and reported pathological response in 44% of patients with 0% pathologic complete response (11, 12). As of late it was discovered that tumours present their driver mutations more poorly on specific MHC molecules in younger and female than in older and male patients (6), showing evidence that presentation of tumour driver mutations varies with sex and age. As such, the response rate to immune checkpoint blockade (ICB) may be dependent on the strength of immune selection occurring early in tumorigenesis (6). In addition, our own work suggests that the pre-existing immunological state is critical to disease progression, mainly dependent on immunological checkpoints such as systemic PD-L1 expression (13). These pathways are amenable to therapeutic intervention with checkpoint inhibitors such as the PD-1 inhibitor Pembrolizumab.

## Case Description

**Figure 1:**
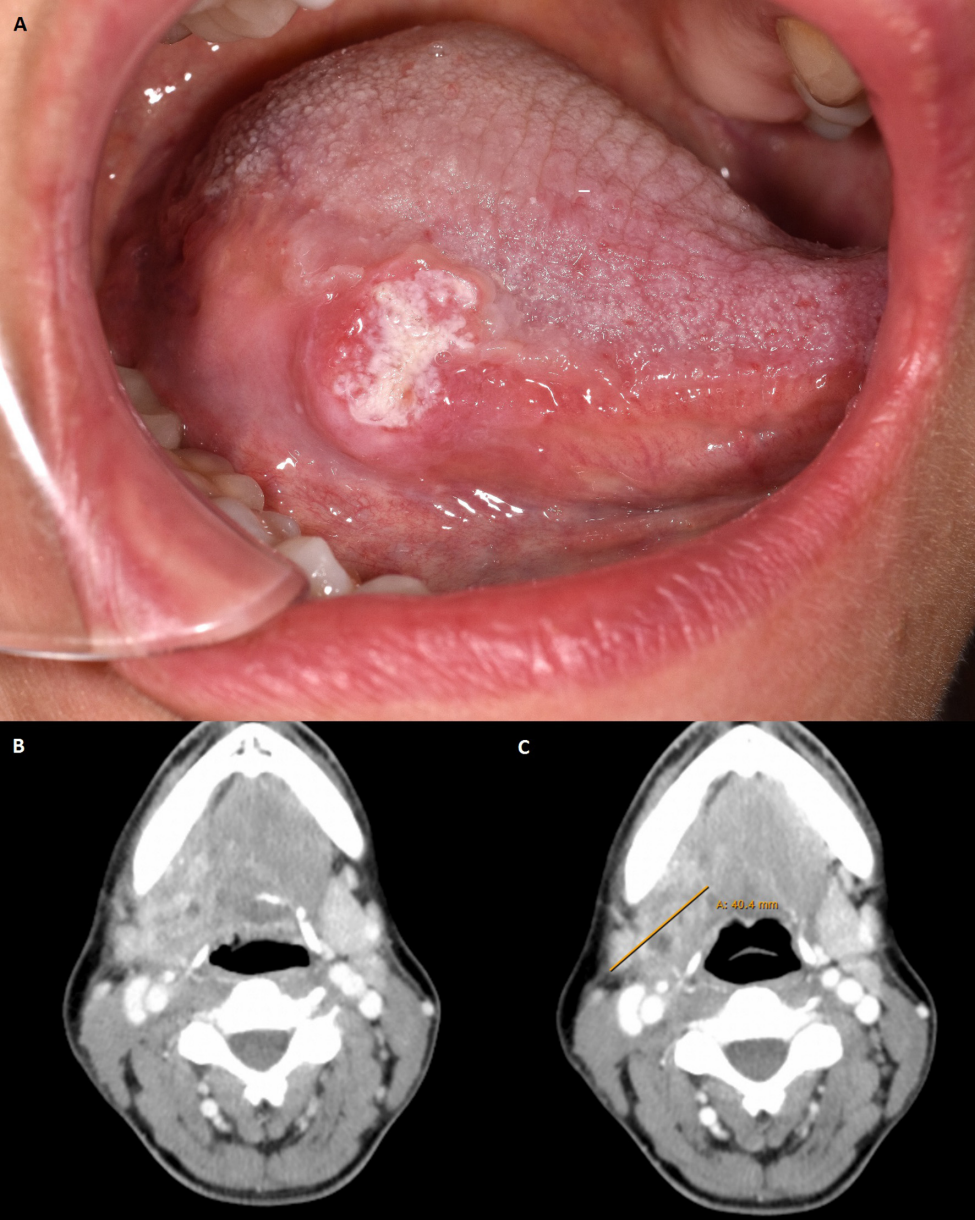
**(A)** Clinical view of primary tumour at initial presentation. Photo taken at the patient’s first presentation. Location of the tumour: right margin of the tongue. **(B)** Computed tomography with suspicious lesion found in tumour follow-up in late 2019. Axial plane with tumour recurrence in the right mandibular angle. **(C)** Computer tomography with pathologically confirmed tumour recurrence after first use of Pembrolizumab 200 mg. Axial plane with tumour recurrence in the right mandibular angle. Max. diameter 40,4 mm. Radiological assessment: Metastasis submandibular increasingly melting.

In mid-2019, a female patient in her late 30’s presented with a squamous cell carcinoma of the oral cavity localized to the right margin of the tongue (**Figure 1A**, **Table 1**). The patient’s medical and family history was unremarkable. Given the patient’s young age, the possibility of fanconi anaemia and dyskeratosis congenita were excluded. The primary tumour was treated by en-bloc resection including transoral hemiglossectomy, partial resection of the floor of mouth and reconstruction by a microvascular radial forearm flap. Selective neck dissection was performed on the right side including level 1-3 (after Robbins, 2002) (14) according to the sequence of resection defined by packages A-E (after Kesting, 2016) (15) with the result of 19 carcinoma-free lymph nodes after pathologic evaluation. The primary tumour was staged pT2 pN0 (0/28) L0 V0 Pn0 G2 R0 (>0.5 cm). Infiltration depth was 0.9 mm. In addition, pathologic evaluation showed no evidence of human papillomavirus association or NUT carcinoma (formerly NUT midline carcinoma). Due to the infiltration depth of 0.9mm adjuvant brachytherapy was performed according to current GEC-ESTRO ACROP recommendations (16). Adjuvant interstitial brachytherapy with 2 doses per day and 3.8 Gy per dose started 2 months and 3 weeks after primary tumour resection and ran for 5 days. Recurrence was diagnosed 5 months after initial surgery and 2 months after completion of brachytherapy and occurred despite initial R0 resection and adjuvant interstitial brachytherapy with 38 Gy total dose. Radiologically, the recurrence appeared as contrast-enhanced lesion on the right submandibular side between the hyoid bone and mandible (**Figure 1B**). It was first discovered 6 months after primary tumour diagnosis during tumour follow-up and confirmed by subsequent biopsy. After review of radiological imaging as well as the clinical situation, the tumour recurrence was classified as marginally resectable because of its hyoid and pharyngeal infiltration and missing infiltration of large neck vessels. Due to the clinical situation and unfavourable prognosis, all therapeutic options were discussed with the patient. Additional to the current standard of care and based on recently published results on the administration of Pembrolizumab prior to surgical therapy (11, 12), the possibility of neoadjuvant and adjuvant immunotherapy as an individual healing attempt was discussed. The patient was informed in detail about the experimental nature of such treatment and the possible risks, including fatal autoimmunologic side effects. The patient was also informed that side effects may occur long after the immunotherapeutic treatment has ended. In addition, she was informed about the rare possibility of hyperprogression and exacerbation of tumour disease due to immunotherapy (17, 18). The need for adequate contraception was pointed out and informed consent was obtained.

**Table 1:** Time history.

Date	Intervention/Event
06/2019	Initial presentation with an OSCC of the right lateral tongue/floor of the mouth
06/2019	Initial surgical tumor treatment:
	Transoral tumor resection with tracheotomy, resection of the right lateral floor of the mouth and right hemi glossectomy, reconstruction with a radial forearm flap, selective neck dissection level 1-3 right (Robbins 2001)
09/2019	Adjuvant radiotherapy as brachytherapy: 3,6 Gy single dose up to 38 Gy total dose
11/2019	Suspect lesion found in tumor aftercare by use of computer tomography
	Biopsy: OSCC
11/2019	Immunotherapy 200 mg Pembrolizumab
12/2019	Surgical tumor treatment of the recurrence:
	Tracheotomy, radical resection of tumor recurrence: partial resection of the dorsal right floor of the mouth, tongue, radial forearm flap in situ, and pharyngeal wall. Partial resection of the right mandibular angle, hyoid bone, and resection of the hypoglossal nerve.
12/2019	Immunotherapy 200 mg Pembrolizumab
01/2020	Immunotherapy 200 mg Pembrolizumab
01/2020-03/2020	Adjuvant chemotherapy and percutaneous radiotherapy Chemotherapy: Cisplatin (40 mg/m2 body surface) in 6 doses Radiotherapy: 2 Gy single dose up to 64 Gy total dose
06/2021	Last computed tomography in mid-2021 with no indication of local recurrence and no lymph node metastases
06/2021	Last tumour follow-up without clinical evidence of local recurrence and without lymph node metastases

Brief summary of the clinical course.

Thereafter, neoadjuvant immunotherapy with 200 mg Pembrolizumab was administered 19 days prior to surgical therapy for the recurrence. The therapy was performed without significant side effects. Shortly before resection of the recurrence, radiologic restaging was performed with respect to neoadjuvant immunotherapy (**Figure 1C**). It showed a slightly reduced progressively melted, and marginally contrast-enhanced lesion in submandibular location on the right. However, compared with the status before Pembrolizumab application, no significant radiological changes were detected (Approximately 1.9 x 2.1 x 2.9 cm compared with 1.5 x 2.2 x 2.2 cm in the previous examination). Surgical therapy was performed according to the state of the art by radical tumour resection with partial resection of the dorsal right floor of the mouth, tongue, radial forearm flap *in situ* and pharyngeal wall. Tumour resection also included partial resection of the right mandibular angle, hyoid bone and resection of the hypoglossal nerve because of its attachment to the tumour mass. The recurrence was staged ypT2 ypN0 (0/2) L0 V0 Pn0 R0 > 5 mm cM0 with an infiltration depth of 6 mm. Defect coverage was accomplished using a microvascular vastus lateralis transplant from the patient’s right thigh. In the postoperative interdisciplinary tumour board, it was decided to re-administer the immunotherapeutic agent according to the patient’s will and to additionally perform an adjuvant radio-chemotherapy according to the current German treatment guideline (19, 20). In accordance with patient will and despite the lack of evidence regarding postoperative adjuvant immunotherapy in OSCC, the immunotherapeutic approach was continued by administering 200 mg of Pembrolizumab, 8 days after recurrence resection. Immunotherapy was continued with the aim of maintaining a constant level of anti-PD1 antibody despite perioperative loss of blood and postoperative application of two units of stored blood. The application had no side effects. Three weeks after the second, a third administration of the immunotherapeutic drug (200 mg Pembrolizumab) was performed without significant side effects. Adjuvant radiochemotherapy was given 7 weeks after surgery over 6 weeks with percutaneous radiotherapy (2 Gy single dose, 64 Gy total dose) and concurrent chemotherapy with cisplatin 40 mg/m2 body surface area in 6 applications. Tumour follow-up took place 6 and 12 months after resection of the tumour recurrence with no evidence of local recurrence (cT0N0). Last computed tomography in mid-2021 with no indication of local recurrence and no lymph node metastases. Regarding the patient’s current clinical condition, wound healing is complete, and the surgical scar is regular. Furthermore, the mouth opening is restricted with an incisal edge difference of 25-30mm. The patient has no tracheostomy, swallowing is possible, but nutrition is supported by a percutaneous feeding tube. Speaking is impaired but possible. The patient is undergoing supportive logopaedic treatment.

## Histopathological Findings

**Figure 2:**
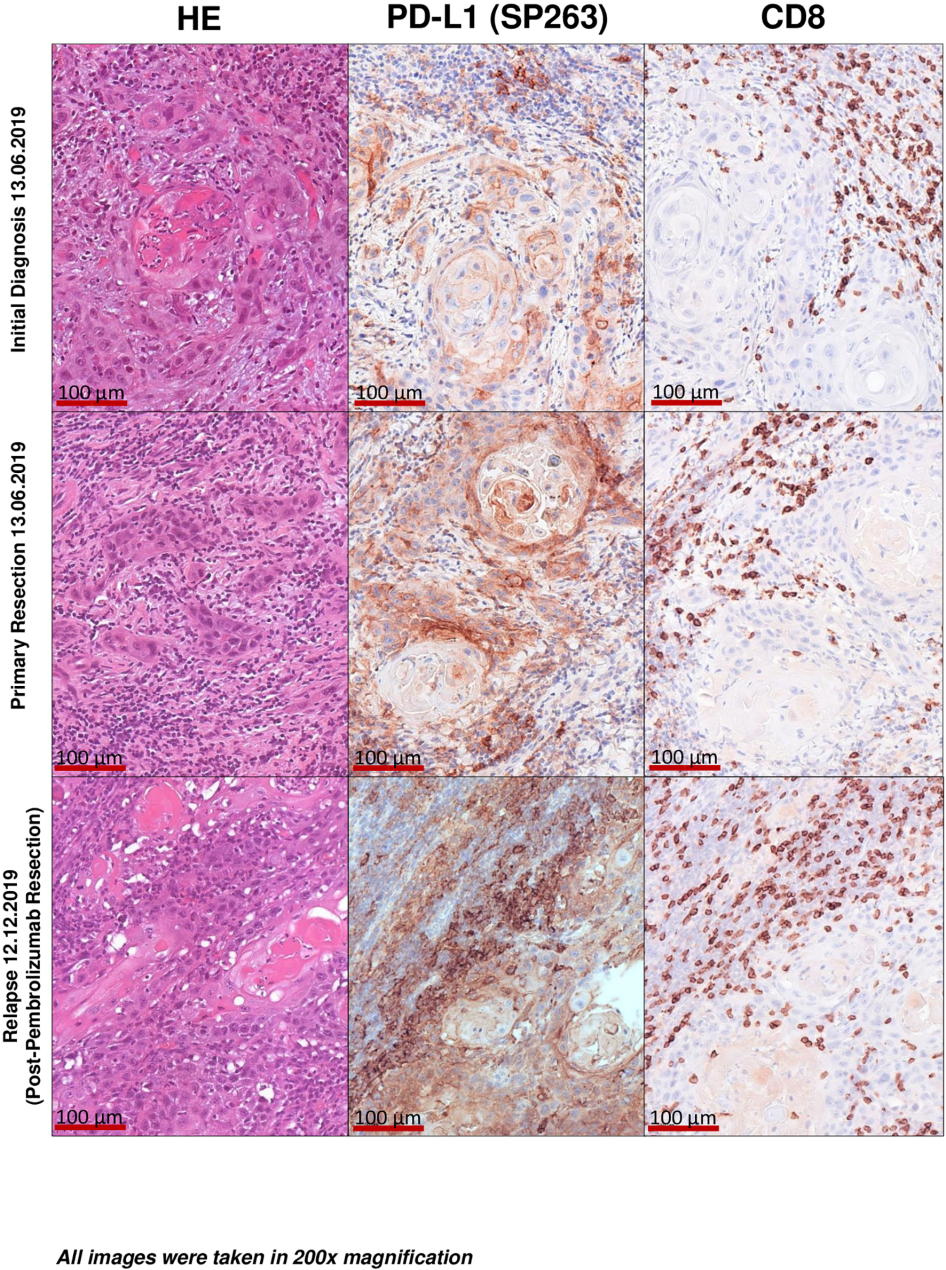
Histopathological imaging. Initial diagnosis. Primary resection. Final post-pembrolizumab resection specimen of tumour recurrence. Images taken at 200× magnification. Histological findings proving a significantly higher CD8+ positive T-Cell density after PD-L1 antibody use in the form of Pembrolizumab may be indicative of the patients significantly improved immunologic status. In addition, continued postoperative use could have a positive impact on the likelihood of a second relapse.

The patient’s tumour presented as moderately differentiated, keratinizing squamous cell carcinoma at initial diagnosis and all further biopsies (G2; **Figure 2** **)**.

**Figure 3:**
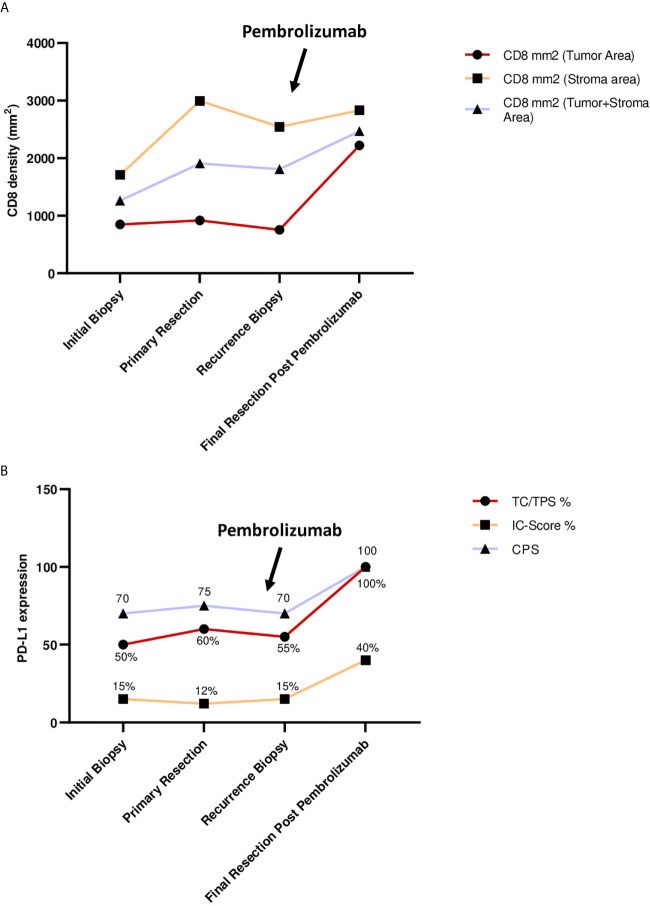
**(A)** CD8 density (mm2) during treatment. Points of interest/sample collection: initial biopsy, primary resection, recurrence biopsy, final resection after Pembrolizumab. Charts show: CD8+ cells per mm2 in tumor area; CD8+ cells per mm2 in stromal area; CD8 mm2 combined tumor and stromal area. **(B)** PD-L1 expression during treatment. Points of interest/sample collection: initial biopsy, primary resection, recurrence biopsy, final resection after Pembrolizumab. Charts show: TC, tumor cells; TPS, tumor proportion score in % (stained tumor cells/tumor cells); IC-Score, inflammatory cell score (stained inflammatory cells/tumor surface); CPS, combined positivity score ((stained tumor cells + stained mononuclear immune cells)/tumor cells), CPS has no dimension.

To further elucidate immunological processes due to Pembrolizumab treatment we applied PD-L1 (Ventana SP263 assay) and CD8 immunohistochemistry to a baseline tumour sample (initial diagnosis), a tumour sample derived from the recurrence before administering Pembrolizumab and the final resection specimen after Pembrolizumab treatment (representative images are depicted in **Figure 3** **)**. The following parameters were chosen for quantification and histological assessment of the conducted therapy: 1. CD8 density per mm2 (Cytotoxic T cell marker) 2. TC/TPS % [TC, tumour cells; TPS, tumour proportion score (stained tumour cells/tumour cells)[ 3. IC-Score % [inflammatory cell score (stained inflammatory cells/tumour surface)] 4. CPS (combined positivity score [(stained tumour cells + stained mononuclear immune cells)/tumour cells)] (21–23). Although the tumour showed a moderate baseline infiltration of CD8+ cytotoxic T-cells (intratumoral CD8+ before pembrolizumab: 756 CD8+ T-cells per mm2) and PD-L1 expression on tumour (TPS 55%, CPS 70) and immune cells (IC-Score 15%), Pembrolizumab treatment led to a significant increase of CD8 infiltration with a very strong increase of intraepithelial localized CD8+ cytotoxic T-cells (intratumoral: 2221 CD8+ T-cells per mm2) and a strong upregulation of PD-L1 expression on tumour cells (TPS 100%, CPS 100) as well as on tumour infiltrating immune cells (IC-Score 40%; **Figures 3A, B** **)**. Furthermore, one cycle of Pembrolizumab was able to induce significant regressive tumour changes. In detail, approximately 75% of the former tumour bed was represented by large fibrotic and necrotic tumour areas while only 25% was covered by residual vital tumour cells. Pre-treatment biopsies showed no tumour regression or necrosis. Taken together, these results indicate that a single cycle of Pembrolizumab treatment can sufficiently booster the pre-existing antitumoral immune response by increasing the ability of cytotoxic effector T-cells to migrate into the tumour microenvironment and to actively kill tumour cells.

## Discussion and Conclusions

The neoadjuvant and adjuvant administration of Pembrolizumab showed no significant side effects. One cycle of Pembrolizumab resulted in significant regressive tumour changes with a large increase in CD8+ immune infiltration, sclerosis, and necrosis of 75% of the tumour mass, as shown by histopathologic assessment. Neither surgical treatment nor adjuvant radiochemotherapy were compromised by the administration of (neo)adjuvant immunotherapy. Although the neoadjuvant use of Pembrolizumab as an individual healing attempt was based on few promising studies and reports in the international literature, in this particular case the patient showed a strong histopathologic response to therapy. At the last follow-up 18 months after salvage surgery, no clinically or radiologically suspicious tumour lesions were detected.

Activation of the co-inhibitory PD-1/PD-L1 pathway is among the mechanisms of OSCC tumour cells to evade immune invasion. Binding of PD-1 on the surface of cytotoxic T cells to PD-L1 or PD-L2 expressed on tumour cells and antigen-presenting cells promotes a microenvironment favourable for tumour growth (24). By this mechanism, the distribution of T cells toward regulatory T cells is dysregulated, while effector T cells and memory T cells are suppressed (25). The concept of PD-1 inhibitors such as Pembrolizumab is based on interrupting the described process to enable an organized immune response against tumour cells. More specifically, inhibition of the PD-1/PD-L1 pathway promotes priming and infiltration of tumour antigen-specific T cells and achieves a relevant tumour necrosis rate before complete surgical ablation of the residual tumour mass (26). Clinical practice for exclusive use of immunotherapy in advanced cases showed a rather low response rate of about 30% (27), optimistically perceived by the oral and maxillofacial surgery community, but still in need of fundamental improvement. Based on our understanding of tumour biology in OSCC, this rate could be increased by using PD-1 inhibitors at an earlier stage of disease, when immunologic conditions are more favourable for the drug to exert its full biologic properties (28). Pembrolizumab is currently approved in Germany only for recurrent and metastatic OSCC when surgical therapy has been exhausted and other conventional treatment options such as radiation or chemotherapy are no longer indicated. At this stage, a significant amount of lymphoid tissue has usually been resected during surgical procedures or otherwise damaged, for example, by the use of radiochemotherapy. Therefore, conditions for therapies that enhance a specific host immune answer against tumour cells are assumed to be significantly deteriorated at that point. However, in a neoadjuvant setting, most of the lymphoid tissue is still intact and therefore the tissue infrastructure might be much more favourable for immunologic cells to infiltrate the tumour. This novel approach could help establish a strong, directed immune response to tumour tissue, including patients with low T-cell infiltration, who are generally associated with poor response rates to immunotherapy (29). Hence, we suggest that neoadjuvant use of PD-1 inhibitors should be further investigated to increase therapeutic efficacy and expand the collective of patients with favourable response to immunotherapy. In a recently published multicentre phase II study, Uppaluri et al. reported the successful neoadjuvant use of Pembrolizumab in addition to standard therapy in patients with locally advanced human papillomavirus-negative head and neck squamous cell carcinoma (HNSCC) (11, 12). All patients received 200 mg neoadjuvant Pembrolizumab and only patients with high-risk pathology (positive margins and/or extranodal extension) received 200 mg adjuvant Pembrolizumab every three weeks for six cycles. The one-year relapse rate among patients with high-risk pathology was only 16.7% (11, 12). Tumour necrosis rates greater than 50% as in our case occurred in 22% of patients and treatment response with ≥10% necrotic tumour cells correlated significantly with initial tumour PD-L1 expression as well as CD8+ cytotoxic T-cell invasion (11, 12). Another ongoing randomized, open-label phase 3 trial called Keynote-689 (NCT03765918) is similarly evaluating the effect of pre- and postoperative use of Pembrolizumab in addition to surgery and standard chemoradiation in patients with previously untreated, resectable, and locally advanced HNSCC (30). While we await the results of this trial, it is expected that the addition of Pembrolizumab will significantly improve pathologic response and event-free survival compared with adjuvant chemoradiotherapy alone. In addition, Pembrolizumab has been shown to be an effective neoadjuvant agent that significantly improves patient survival in other cancers, including malignant melanoma, bladder cancer and lung cancer (31–33). Phase III trials in various solid cancers are currently underway to confirm the results of the present preliminary studies in a large patient population (NCT03036488, NCT03221426). The potential benefit of neoadjuvant Pembrolizumab administration can be assessed by histopathologic changes in the patient’s tumour progression (**Figure 2**). The patient in this case already showed relatively strong PD-L1 and CD8 expression before the use of immunotherapy, as previously described as a typical feature in non-smoking and nondrinking patients with OSCC (34). However, after one dose of neoadjuvant Pembrolizumab, CD 8 expression increased markedly, particularly in the tumour tissue region, where the density of CD8+ T cells more than doubled. This can be interpreted as a clear sign of specific immune cell infiltration into the tumour, which can then lead to targeted lysis of tumour cells as seen in the histologic specimen at recurrence. Similar observations were made for PD-L1 expression, which drastically increased after neoadjuvant Pembrolizumab application as shown in **Figures 3A, B**. Both high PD-L1 expression and high CD8 expression were previously described as factors associated with positive response to PD1/PD-L1 inhibitor therapy in OSCC (35). The positive predictions can be confirmed by the case described: a single dose of neoadjuvant Pembrolizumab resulted in significant necrosis of tumour tissue, although no radiologic correlate to the remarkable therapeutic response was detectable. This may be due to the fact that a certain number of patients treated with immunotherapy show atypical radiologic response patterns compared with other conventional treatment modalities, making it difficult to quantify the efficacy of immunotherapy (36). Because of the patient’s unfavourable prognosis due to early recurrence, our interdisciplinary tumour board recommended neoadjuvant immunotherapy as an individual healing attempt in addition to the current standard of care. Neoadjuvant Pembrolizumab was successfully administered 19 days before surgical therapy for recurrence in OSCC. Immunotherapy was continued adjuvant, although R0 resection was achieved and no extranodal tumour extension was detected on histopathologic evaluation. This was attributed to the fact that ablative tumour surgery is associated with relevant blood loss and, therefore, the plasma concentration of Pembrolizumab could decrease significantly after tumour resection. However, to our knowledge, there is no explicit clinical evidence to date demonstrating that immunotherapy is beneficial for patients with OSCC when complete tumour resection has been achieved. Furthermore, it was the patients’ will to continue Pembrolizumab therapy after tumour surgery, as the single preoperative administration resulted in a clearly histologically measurable attenuation of the tumour mass. This case demonstrates that neoadjuvant immunotherapy with Pembrolizumab can be performed for recurrent or advanced OSCC without delaying or complicating subsequent treatment, and that the therapeutic benefit of (neo)adjuvant immunotherapy is more extensive than a salvage option when all other therapeutic alternatives have been exhausted. However, a detailed clinical recommendation for pre-selection of patients eligible for Pembrolizumab therapy, including timing, dosing, and number of cycles of neoadjuvant as well as adjuvant administration, is still pending. Further studies should be conducted in future trials to evaluate patient eligibility criteria and treatment monitoring options for neoadjuvant use of Pembrolizumab in OSCC.

## Data Availability Statement

The original contributions presented in the study are included in the article/supplementary material. Further inquiries can be directed to the corresponding author.

## Author Contributions

MW, MK, RL, GF, MH, T-OB, and JG examined and treated the patient and collected the data. ME participated in the pathological diagnosis. MO, MW, and JG discussed the case and data. MO, MW, and JG wrote the manuscript. JR, RL, GF, and T-OB aided with manuscript writing and language prettification. All authors contributed to the article and approved the submitted version.

## Conflict of Interest

The authors declare that the research was conducted in the absence of any commercial or financial relationships that could be construed as a potential conflict of interest.

## Abbreviations

OSCC, oral squamous cell carcinoma; R0 resection, no cancer cells seen microscopically at the primary tumor site; PD-L1, programmed cell death 1 ligand 1; MHC, major histocompatibility complex; PD-1, programmed cell death protein 1; ICB, Immune checkpoint blockade; CD8, Cytotoxic T cell marker; TC, tumor cells; TPS, tumor proportion score; IC-score, inflammatory cell score; CPS, combined positivity score; HNSSC, head and neck squamous cell carcinoma; HE, hematoxylin-eosin-stain.

## References

[1] Warnakulasuriya S. Causes of Oral Cancer–an Appraisal of Controversies. Br Dent J (2009) 207(10):471–5. 10.1038/sj.bdj.2009.1009 19946320

[2] Mortazavi H Baharvand M Mehdipour M. Oral Potentially Malignant Disorders: An Overview of More Than 20 Entities. J Dent Res Dent Clin Dent Prospects (2014) 8(1):6–14. 10.5681/joddd.2014.002 25024833 PMC4091702

[3] Liao CT Chang JT Wang HM Ng SH Hsueh C Lee LY. Salvage Therapy in Relapsed Squamous Cell Carcinoma of the Oral Cavity: How and When? Cancer (2008) 112(1):94–103. 10.1002/cncr.23142 18022917

[4] Montero PH Patel SG. Cancer of the Oral Cavity. Surg Oncol Clin N Am (2015) 24(3):491–508. 10.1016/j.soc.2015.03.006 25979396 PMC5018209

[5] Schreiber RD Old LJ Smyth MJ. Cancer Immunoediting: Integrating Immunity’s Roles in Cancer Suppression and Promotion. Science (2011) 331(6024):1565–70. 10.1126/science.1203486 21436444

[6] Castro A Pyke RM Zhang X Thompson WK Day CP Alexandrov LB. Strength of Immune Selection in Tumors Varies With Sex and Age. Nat Commun (2020) 11(1):4128. 10.1038/s41467-020-17981-0 32807809 PMC7431859

[7] Kujan O van Schaijik B Farah CS. Immune Checkpoint Inhibitors in Oral Cavity Squamous Cell Carcinoma and Oral Potentially Malignant Disorders: A Systematic Review. Cancers (Basel) (2020) 12(7):1. 10.3390/cancers12071937 PMC7409293 32708945

[8] Nosrati A Tsai KK Goldinger SM Tumeh P Grimes B Loo K. Evaluation of Clinicopathological Factors in PD-1 Response: Derivation and Validation of a Prediction Scale for Response to PD-1 Monotherapy. Br J Cancer (2017) 116(9):1141–7. 10.1038/bjc.2017.70 PMC5418446 28324889

[9] Migden MR Rischin D Schmults CD Guminski A Hauschild A Lewis KD. PD-1 Blockade With Cemiplimab in Advanced Cutaneous Squamous-Cell Carcinoma. N Engl J Med (2018) 379(4):341–51. 10.1056/NEJMoa1805131 29863979

[10] Economopoulou P Perisanidis C Giotakis EI Psyrri A. The Emerging Role of Immunotherapy in Head and Neck Squamous Cell Carcinoma (HNSCC): Anti-Tumor Immunity and Clinical Applications. Ann Transl Med (2016) 4(9):173. 10.21037/atm.2016.03.34 27275486 PMC4876265

[11] Uppaluri R Campbell KM Egloff AM Zolkind P Skidmore ZL Nussenbaum B. Neoadjuvant and Adjuvant Pembrolizumab in Resectable Locally Advanced, Human Papillomavirus-Unrelated Head and Neck Cancer: A Multicenter, Phase II Trial. Clin Cancer Res (2020) 26(19):5140–52. 10.1158/1078-0432.CCR-20-1695 PMC7547532 32665297

[12] Uppaluri R Campbell KM Egloff AM Zolkind P Skidmore ZL Nussenbaum B. Correction: Neoadjuvant and Adjuvant Pembrolizumab in Resectable Locally Advanced, Human Papillomavirus-Unrelated Head and Neck Cancer: A Multicenter, Phase II Trial. Clin Cancer Res (2021) 27(1):357. 10.1158/1078-0432.CCR-20-4484 33397681

[13] Weber M Wehrhan F Baran C Agaimy A Büttner-Herold M Preidl R. PD-L1 Expression in Tumor Tissue and Peripheral Blood of Patients With Oral Squamous Cell Carcinoma. Oncotarget (2017) 8(68):112584–97. 10.18632/oncotarget.22576 PMC5762533 29348848

[14] Robbins KT Clayman G Levine PA Medina J Sessions R Shaha A. Neck Dissection Classification Update: Revisions Proposed by the American Head and Neck Society and the American Academy of Otolaryngology-Head and Neck Surgery. Arch Otolaryngol Head Neck Surg (2002) 128(7):751–8. 10.1001/archotol.128.7.751 12117328

[15] Koerdt S Röckl J Rommel N Mücke T Wolff KD Kesting MR. Lymph Node Management in the Treatment of Oral Cancer: Analysis of a Standardized Approach. J Craniomaxillofac Surg (2016) 44(10):1737–42. 10.1016/j.jcms.2016.08.002 27580851

[16] Kovács G Martinez-Monge R Budrukkar A Guinot JL Johansson B Strnad V. GEC-ESTRO ACROP Recommendations for Head & Neck Brachytherapy in Squamous Cell Carcinomas: 1st Update - Improvement by Cross Sectional Imaging Based Treatment Planning and Stepping Source Technology. Radiother Oncol (2017) 122(2):248–54. 10.1016/j.radonc.2016.10.008 27889184

[17] Frelaut M Le Tourneau C Borcoman E. Hyperprogression Under Immunotherapy. Int J Mol Sci (2019) 20(11):10f. 10.3390/ijms20112674 PMC6600249 31151303

[18] Adashek JJ Kato S Ferrara R Lo Russo G Kurzrock R. Hyperprogression and Immune Checkpoint Inhibitors: Hype or Progress? Oncologist (2019) 25:94–8. 10.1634/theoncologist.2019-0636 PMC7011624 32043794

[19] Frerich B. Standardtherapie Von Plattenepithelkarzinomen Der Mundhöhle Gemäß Leitlinien. Der MKG-Chirurg (2018) 11(1):5–14. 10.1007/s12285-018-0152-7

[20] Available at: https://www.awmf.org/uploads/tx_szleitlinien/007-100OLl_S3_Mundh%C3%B6hlenkarzinom_122012-122015-abgelaufen.pdf.

[21] Witte HM Gebauer N Lappöhn D Umathum VG Riecke A Arndt A. Prognostic Impact of PD-L1 Expression in Malignant Salivary Gland Tumors as Assessed by Established Scoring Criteria: Tumor Proportion Score (TPS), Combined Positivity Score (CPS), and Immune Cell (IC) Infiltrate. Cancers (Basel) (2020) 12(4):13f. 10.3390/cancers12040873 PMC7226358 32260165

[22] Shimizu S Hiratsuka H Koike K Tsuchihashi K Sonoda T Ogi K. Tumor-Infiltrating CD8(+) T-Cell Density Is an Independent Prognostic Marker for Oral Squamous Cell Carcinoma. Cancer Med (2019) 8(1):80–93. 10.1002/cam4.1889 30600646 PMC6346233

[23] Schildhaus HU. [Predictive Value of PD-L1 diagnostics]. Pathologe (2018) 39(6):498–519. 10.1007/s00292-018-0507-x 30367225

[24] Ferris RL. Immunology and Immunotherapy of Head and Neck Cancer. J Clin Oncol (2015) 33(29):3293–304. 10.1200/JCO.2015.61.1509 PMC4586169 26351330

[25] Zou W Chen L. Inhibitory B7-Family Molecules in the Tumour Microenvironment. Nat Rev Immunol (2008) 8(6):467–77. 10.1038/nri2326 18500231

[26] Bardhan K Anagnostou T Boussiotis VA. The PD1:PD-L1/2 Pathway From Discovery to Clinical Implementation. Front Immunol (2016) 7:550. 10.3389/fimmu.2016.00550 28018338 PMC5149523

[27] Thommen DS Schumacher TN. T Cell Dysfunction in Cancer. Cancer Cell (2018) 33(4):547–62. 10.1016/j.ccell.2018.03.012 PMC7116508 29634943

[28] Gonzalez H Hagerling C Werb Z. Roles of the Immune System in Cancer: From Tumor Initiation to Metastatic Progression. Genes Dev (2018) 32(19-20):1267–84. 10.1101/gad.314617.118 PMC6169832 30275043

[29] Tumeh PC Harview CL Yearley JH Shintaku IP Taylor EJ Robert L. PD-1 Blockade Induces Responses by Inhibiting Adaptive Immune Resistance. Nature (2014) 515(7528):568–71. 10.1038/nature13954 PMC4246418 25428505

[30] Uppaluri R Lee NY Westra W Cohen EEW Haddad RI Temam S. KEYNOTE-689: Phase 3 Study of Adjuvant and Neoadjuvant Pembrolizumab Combined With Standard of Care (SOC) in Patients With Resectable, Locally Advanced Head and Neck Squamous Cell Carcinoma. J Clin Oncol (2019) 37(15_suppl):TPS6090–TPS. 10.1200/JCO.2019.37.15_suppl.TPS6090

[31] Huang AC Orlowski RJ Xu X Mick R George SM Yan PK. A Single Dose of Neoadjuvant PD-1 Blockade Predicts Clinical Outcomes in Resectable Melanoma. Nat Med (2019) 25(3):454–61. 10.1038/s41591-019-0357-y PMC6699626 30804515

[32] Rouanne M Bajorin DF Hannan R Galsky MD Williams SB Necchi A. Rationale and Outcomes for Neoadjuvant Immunotherapy in Urothelial Carcinoma of the Bladder. Eur Urol Oncol (2020) 3(6):728–38. 10.1016/j.euo.2020.06.009 33177001

[33] Hill A Gong J Wilczynski S Mirza R Erhunmwunsee L Salgia R. Complete Pathologic Response When Adding Pembrolizumab to Neoadjuvant Chemotherapy in Stage IIIA Non-Small-Cell Lung Cancer. J Oncol Pract (2018) 14(9):569–71. 10.1200/JOP.18.00127 30044685

[34] Foy JP Bertolus C Michallet MC Deneuve S Incitti R Bendriss-Vermare N. The Immune Microenvironment of HPV-Negative Oral Squamous Cell Carcinoma From Never-Smokers and Never-Drinkers Patients Suggests Higher Clinical Benefit of IDO1 and PD1/PD-L1 Blockade. Ann Oncol (2017) 28(8):1934–41. 10.1093/annonc/mdx210 28460011

[35] Machiels JH Coulie PG. The Promise of Immunostimulatory Antibodies in Head and Neck Cancer. Lancet Oncol (2016) 17(7):856–7. 10.1016/S1470-2045(16)30106-1 27247227

[36] Doumas S Foukas PG Economopoulou P Kotsantis I Psyrri A. Atypical Patterns of Responses in the Era of Immune Checkpoint Inhibitors in Head and Neck Cancer. Oral Oncol (2020) 100:104477. 10.1016/j.oraloncology.2019.104477 31837533

